# Evaluation of inter-departmental variability of ejection fraction and cardiac volumes in myocardial perfusion scintigraphy using simulated data

**DOI:** 10.1186/s40658-014-0105-9

**Published:** 2015-01-23

**Authors:** Elin Trägårdh, Michael Ljungberg, Lars Edenbrandt, Eva Örndahl, Lena Johansson, Agneta Gustafsson, Cathrine Jonsson, Jessica Hagerman, Katrine Riklund, David Minarik

**Affiliations:** Clinical Physiology and Nuclear Medicine, Skåne University Hospital, Lund University Hospital, Inga Marie Nilssons gata 49, 205 02 Malmö, Sweden; Department of Medical Radiation Physics, Lund University, Lund, Sweden; Equalis AB, Uppsala, Sweden; Clinical Physiology, Central Hospital, Karlstad, Sweden; Department of Medical Physics, Karolinska University Hospital, Stockholm, Sweden; Department of Radiation Sciences, Umeå University, Umeå, Sweden; Radiation Physics, Skåne University Hospital, Lund University, Malmö, Sweden

**Keywords:** External quality assessment, Monte Carlo simulations, SPECT, Myocardial perfusion imaging

## Abstract

**Background:**

Myocardial perfusion scintigraphy (MPS) is a clinically useful noninvasive imaging modality for diagnosing patients with suspected coronary artery disease. By utilizing gated MPS, the end diastolic volume (EDV) and end systolic volume (ESV) can be measured and the ejection fraction (EF) calculated, which gives incremental prognostic value compared with assessment of perfusion only. The aim of this study was to evaluate the inter-departmental variability of EF, ESV, and EDV during gated MPS in Sweden.

**Methods:**

Seventeen departments were included in the study. The SIMIND Monte Carlo (MC) program together with the XCAT phantom was used to simulate three patient cases with different EDV, ESV, and EF. Individual simulations were performed for each department, corresponding to their specific method of performing MPS. Images were then sent to each department and were evaluated according to clinical routine. EDV, ESV, and EF were reported back.

**Results:**

There was a large underestimation of EDV and ESV for all three cases. Mean underestimation for EDV varied between 26% and 52% and for ESV between 15% and 60%. EF was more accurately measured, but mean bias still varied between an underestimation of 24% to an overestimation of 14%. In general, the intra-departmental variability for EDV, ESV, and EF was small, whereas inter-departmental variability was larger.

**Conclusions:**

Left ventricular volumes were generally underestimated, whereas EF was more accurately estimated. There was, however, large inter-departmental variability.

## Background

Myocardial perfusion scintigraphy (MPS) is widely regarded as a clinically useful non-invasive imaging modality for diagnosing patients with suspected coronary artery disease. The diagnostic accuracy is high, and risk stratification has been well validated [1-3]. By utilizing gated MPS, the end diastolic volume (EDV) and end systolic volume (ESV) can be measured and the ejection fraction (EF) can thus be calculated. Assessment of left ventricular volumes and function are important parameters in patients with coronary artery disease. The information from gated MPS has been shown to have incremental prognostic value compared with assessment of perfusion only [4,5]. Decreased EF and increased left ventricular volumes without any perfusion defects are seen in, for example, some cases of cardiomyopathy. A stress perfusion defect with decreased function compared with the resting images is indicative of postischemic myocardial stunning. Lower EF and larger volumes after stress compared with rest are signs of severe ischemia.

It is vital that different departments measure the same EF and left ventricular volumes. The aim of this study was to evaluate the inter-departmental variability of EF, ESV, and EDV obtained from gated MPS in Sweden. The study was performed as a part of a national QA program in nuclear medicine, initiated and managed by Equalis AB [6], a non-profit company providing external quality assessment of laboratory investigations within the Swedish healthcare.

Evaluation of inter-departmental variability of EF, ESV, and EDV has been performed by Verberne et al. [7]. They used a physical phantom [8], which had a dynamic heart and modifiable ESV and EDV. This method of evaluation requires that each participating department owns its own phantom or that one or more phantoms are sent around. Each department also has to perform several measurements including phantom preparation that requires both time and effort. To overcome the inconvenience of constructing and sending a physical phantom around, we have instead created Monte Carlo simulated gated MPS studies for distribution. By using the Monte Carlo method, we are able to use a more realistic digital phantom with more degrees of freedom regarding the shape of the phantom, physiological movements due to breathing, and the motion of the heart.

## Methods

Twenty-six departments were enrolled to participate in the evaluation. Four were not able to participate because they either use the new CZT cameras or use special heart collimators which at the moment are not supported by the Monte Carlo program. Five departments did not respond/had troubles reading the data files. Seventeen departments ended up participating in the evaluation.

Each participating department reported the method they use to perform MPS, e.g. the camera system, camera settings (energy window, matrix size, number of projection angles, etc.), and the administered radioactivity. All reported parameters are listed in Table 1. The SIMIND Monte Carlo program [9] together with the XCAT anthropomorphic computer phantom [10] was then used to simulate projection data. Simulations were performed for each department using their characteristic camera settings. Three different patient cases were simulated as described in Table 2. The cases were chosen to cover as much of the patient spectrum that is normally encountered in the clinic with the three cases. For each patient case, 32 instances of the XCAT phantom were created with the heart in different positions in the cardiac cycle and different spatial positions in the thoracic cavity due to breathing. For each department, all the 32 phantoms were simulated and the results were summed corresponding to each department’s method of gating, i.e. if 8 or 16 gates were used. The biokinetics was taken from [11], and the fraction of the administered activity in the heart after 1 h was set to 1.2%. Here, we did not take into account eventual differences between tetrofosmin and sestamibi. In order to mimic real measurements, the simulations were performed with sufficient histories to generate noiseless data. Poisson noise was then added after the simulations, corresponding to a count level representative for the administered activity and acquisition time for each department and the aforementioned biokinetics. The final projections were converted to DICOM format and sent to the departments, which evaluated the simulations as according to their methods of tomographic reconstruction and ESV, EDV, and EF quantifications. The departments used different software packages for the evaluation, according to clinical routine (reported use of software packages for different departments: Quantitative Gated SPECT (Cedars Sinai Los Angeles, CA, USA; QPS), AutoQuant (Philips, Andover, MA, USA), EXINI Heart (EXINI Diagnostics, Lund, Sweden), Emory Cardiac Toolbox (Emory University Medical Center, Atlanta, GA, USA)). The evaluation was restricted to non-attenuation corrected images and reconstruction without collimator detector response compensations because of difficulties of generating DICOM images with the required header information for each gamma camera vendor. The methods used by the departments are listed in Table 3; however, some departments did not report their methods. Five departments contributed with one response each and 12 departments contributed with multiple responses since more than one technologist performed the evaluation.

**Table 1:** **Parameters that were provided from the different departments for the simulations**

**Camera system**	**Starting angle**
Collimator	Total rotation
Crystal thickness	Number of projections
Energy window	Time per projection
Matrix size	Number of time frames
Pixel size	Administered activity

**Table 2:** **Patient characteristics for the three cases**

	**Case 1**	**Case 2**	**Case 3**
Sex	Female	Male	Female
Length (cm)	160	182	171
Weight (kg)	55	102	68
EF (%)	53	37	62
EDV (mL)	50	230	91
ESV (mL)	24	143	35

**Table 3:** **Reconstruction algorithm and evaluation software that were reported from the departments**

**Department number**	**Reconstruction algorithm**	**Iteration updates**	**Filter**	**Cut-off frequency/width** ^**a**^	**Evaluation software**
1	N/A				
2	FBP		Butterworth	0.52	ECToolbox
3	OSEM	80	Butterworth	0.45	QGS
4	OSEM	N/A	Hanning		QGS
5	OSEM	48	Butterworth	0.4	AutoQuant
6	N/A				
7	FBP		Butterworth	0.35	QGS
8	N/A				
9	FBP		Butterworth	0.35	Exini heart
10	FBP		Butterworth	0.65	QGS
11	N/A				
12	FBP		Butterworth	0.4	QGS
13	FBP		Butterworth	0.9	QGS
14	FBP		Butterworth	0.52	QGS
15	OSEM	120	Butterworth	0.4	QGS
16	FBP		Butterworth	0.4	ECtoolbox
17	OSEM	32	3D Gaussian	0.8	QGS

^**a**^Cut-off frequency for Butterworth filter is in unit of Nyquist frequency and SD for 3D Gaussian filter is in pixels.

*FBP* filtered back projection, *OSEM* ordered subset expectation maximization.

### Statistical analysis

Values of reported EDV, ESV, and EF are given as mean and 95% confidence interval (CI). Mean bias as well as mean absolute bias was calculated. One sample *t*-test was used to determine differences between reported values and the true simulated value for EDV, ESV, and EF. When more than one value was reported from one department, the mean value from that department was used in the *t*-test. Statistical significance was set at 0.05. Statistical analysis was carried out using MedCalc for Windows, version 12.7.7.0 (MedCalc Software, Ostend, Belgium).

## Results

**Figure 1:**
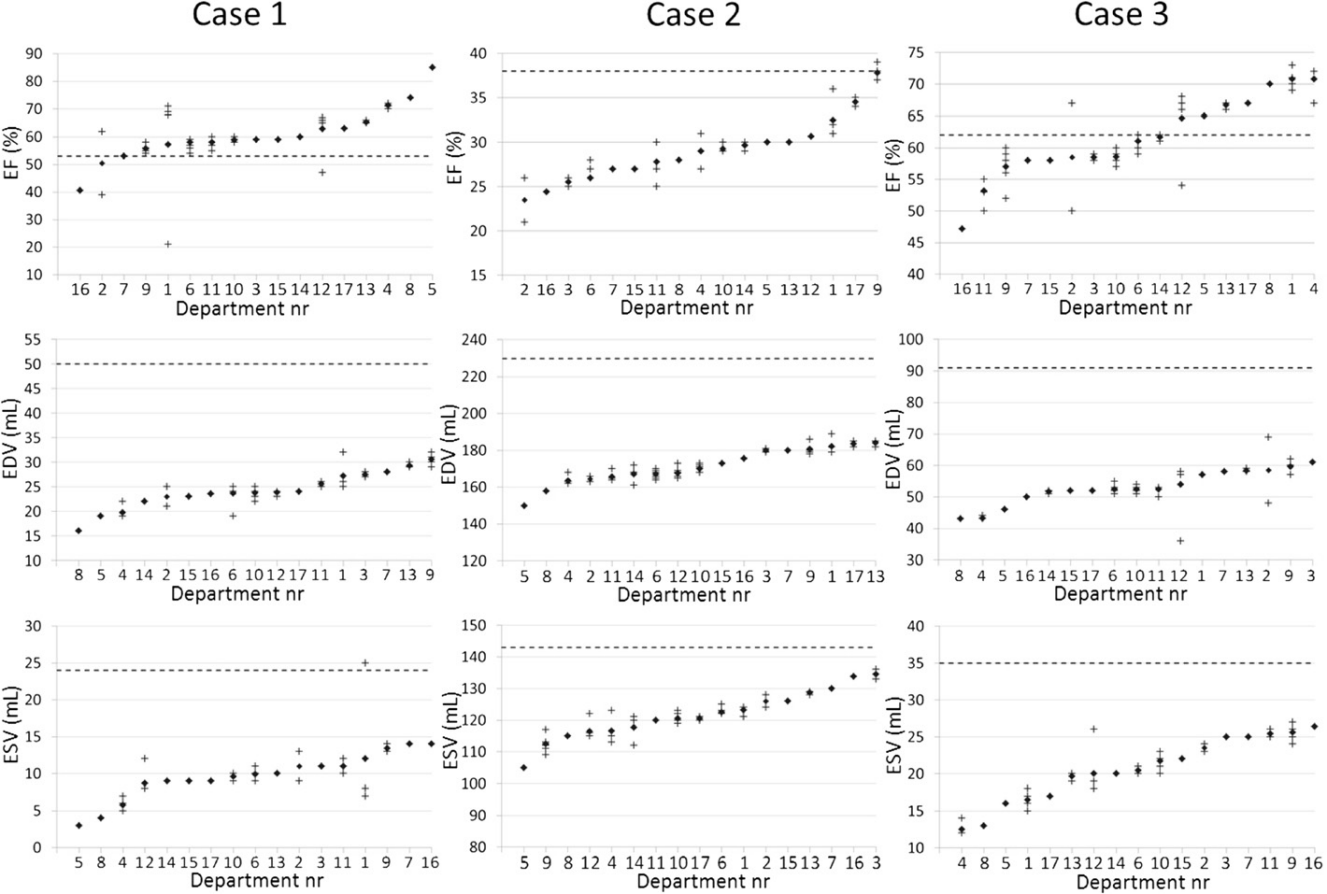
**Dotted lines represent true values.** Plus signs represent answers from individual technologists (provided by 12 departments) and diamond signs represent the department mean.

The results are displayed in Figure 1 and Table 4. There was a large underestimation of both EDV and ESV for all three cases. The largest underestimation was seen for case 1 (female with a small heart) and the smallest underestimation for case 2 (male with a large heart). Mean underestimation for EDV varied between 26% and 52% and for ESV between 15% and 60%. EF was more accurately estimated, but mean bias still varied between an underestimation of 24% to an overestimation of 14%. The mean absolute bias was 17%, 24%, and 9% for cases 1 to 3, respectively. In general, the intra-departmental variability for EDV, ESV, and EF was small, whereas inter-departmental variability was larger (Figure 1). The outlier in department no. 1 could be due to a typo.

**Table 4:** **Mean values, 95% CI, mean bias, and** ***p*** **values for all departments**

	**Case 1**			**Case 2**			**Case 3**		
	*EF*	*EDV*	*ESV*	*EF*	*EDV*	*ESV*	*EF*	*EDV*	*ESV*
Mean all departments (mL)	61	24	10	29	171	122	62	53	21
95% CI (mL)	42 to 79	17 to 31	4 to 16	22 to 36	153 to 190	107 to 136	49 to 74	43 to 63	12 to 29
Mean bias (%)	14	−52	−60	−24	−26	−15	−1	−42	−41
*p* value	0.005	<0.001	<0.001	<0.001	<0.001	<0.001	0.77	<0.001	<0.001

## Discussion

Our study shows that there is a large inter-departmental variability of EF, EDV, and ESV estimations in nuclear medicine departments in Sweden, but the intra-departmental variability was found to be small. We found a large underestimation of EDV and ESV, but since the underestimation of EDV and ESV was of similar order, the EF estimates were more accurate, although not satisfactory. The mean bias varied from 14% for the small heart, where 14 out of 17 departments overestimated the EF, to −24% for the large heart, where all but one underestimated the EF. For the normal heart, the mean bias was around 1% but the mean absolute bias was however 9%, indicating that the accuracy of the EF estimates also for the normal sized heart was low. Since the evaluation of EF to a large extent is automated, there should be a small intra-departmental variability and we are recommending that departments that have a larger intra-departmental variability should perform an internal quality control of their method. The large inter-departmental variability is however of concern, both for cardiac volumes and for EF. The volume estimates of the LV depend on the spatial resolution of the final images and the algorithm used to outline the LV. The spatial resolution depends on several parameters such as choice of collimator, pixel size, noise level, which determines eventual pre- or post-reconstruction filters, and the reconstruction algorithm. The noise level in turn is dependent on administered activity, measurement time per frame, number of frames per heart cycle, number of projections, and pixel size. Different software packages use different algorithms to outline the LV, and these are more or less prone to give different results depending on the resolution in the input image. It was beyond the scope of this study to evaluate to what extent each parameter affects the final result because the statistical basis was too small. However, some tendencies can be extracted from the data. It can be seen that ECToolbox generally gave lower EF than other softwares. Also, departments using OSEM or FBP with higher cut-off frequency gave more accurate volume estimates of the small heart due to better resolution. Regarding the cases with normal and large heart, the results were more arbitrary, indicating that it is a combination of measurement protocol, reconstruction parameters, and evaluation software that determine the outcome of the volume estimates.

In the study by Verberne et al. [7], they used a physical phantom that included a dynamic heart model able to define different ESV, EDV, and heart wall thickness but the phantom could not mimic physical properties such as heart motion due to breathing. This phantom was distributed to 35 nuclear medicine departments for individual measurements using three different settings; with an ESV of 50, 90, and 120 mL respectively and an EDV of 120, 160, and 190 mL respectively, which give an EF of 58%, 44%, and 37%, respectively. Results showed that the EDV and ESV were on average underestimated by 1 to 65 mL respectively and the EF was overestimated by 1% to 15%, while our results showed both under- and overestimation of the EF. This difference was probably due the fact that we had a more differentiated phantom population. Also, in their study, only the QGS software package was used to quantify the measurements; whereas in our study, the contributing departments used their clinical routine software packages.

Several previous studies have compared LV volumes as quantified by different algorithms to cardiac magnetic resonance imaging, regarded as the reference standard. In the majority of the studies, EDV and ESV by MPS were underestimated [12-18]. EDV by MPS has also been shown to range from overestimation to underestimation compared to magnetic resonance imaging depending on the software package used [19-21]. One study has shown an overestimation in both EDV and ESV by MPS [22]. An explanation for the differences found in these studies, apart from differences in the algorithms for calculating MPS volumes, could be different magnetic resonance sequences. It has been shown that older turbo gradient echo imaging shows significantly smaller EDV and ESV compared to current standard steady-state free precession imaging [23]. The results obtained from the studies using newer magnetic resonance imaging technique are in accordance with our results.

Based on the results of our and others studies, it should be debated whether ESV and EDV should be provided in a clinical final report of the MPS. The American Society of Nuclear Cardiology conclude in their consensus statement [24] on reporting of myocardial perfusion imaging studies that a quantitative value of left ventricular EF should be included within the report. For EF greater than 60%, the actual calculated number should be included in the report and mention made of overestimation in patients with small hearts. Ventricular volumes may be reported (optional). The European Association of Nuclear Medicine and European Society of Cardiology state in their procedural guidelines for myocardial perfusion imaging [25] that EF and volumes should be reported together and that caution should be exercised in reporting apparently spurious values of these parameters. It is also stated that reporting of volumes may preferably be indexed according to body surface area, since the reference values have a narrower range. We believe that if physicians want to state estimated EF and volumes in their reports, they should be careful to use normal limits that have been established for the same MPS technique and software tool as used at their department.

This study has some limitations. One is that we only enrolled departments that use standard gamma cameras with parallel hole collimators. Newer technologies, such as the new semiconductor cameras (CZT cameras) and special collimators, such as the IQ-SPECT system (Siemens, Erlangen, Germany) could not be simulated in the present version of the SIMIND code. Also, only non-attenuation corrected images were used, since departmental specific attenuation maps generated from CT were difficult to create. Thus, the results from the study do not exactly represent the clinical situation in Sweden but indicate tendencies of the large variability. The SIMIND Monte Carlo program is a useful tool to test an imaging method when the aim is to test the robustness of the method in a patient-like situation. However, a simulation is not entirely equal to a real measurement since the effects of e.g. non-perfect intrinsic spatial uniformity and linearity is not modeled. Also, eventual patient movement in addition to breathing and beating heart is not modeled. The heart in the phantoms used in the study is derived from one real patient and scaled to different sizes. Although attenuation conditions such as amount of fat, size of the heart, and breasts differ, the shape of the heart is the same in all cases.

## Conclusion

In our study, we have used the MC method for a multi-center quality control study of MPI, both in terms of differences between departments and the general accuracy of the method. We have confirmed the results from previous studies that left ventricular volumes are underestimated in MPS, whereas EF is more accurately estimated. There was, however, large inter-departmental variability, which needs to be further addressed.
